# Survival and prognostic analysis of T-cell lymphoblastic lymphoma patients treated with dose-adjusted BFM-90 regimen

**DOI:** 10.18632/aging.204008

**Published:** 2022-04-10

**Authors:** Hui Yu, Lan Mi, Fei Qi, Xing Wang, Yingying Ye, Miaomiao Li, Dedao Wang, Ning Ding, Xiaogan Wang, Yuqin Song, Jun Zhu, Yan Xie

**Affiliations:** 1 Key Laboratory of Carcinogenesis and Translational Research, Ministry of Education, Department of Lymphoma, Peking University Cancer Hospital and Institute, Beijing, China

**Keywords:** T-cell lymphoblastic lymphoma, BFM-90, conditional survival, neutrophil-to-lymphocyte ratio

## Abstract

We aimed to investigate the long-term prognosis and prognostic factors of T-cell lymphoblastic lymphoma (T-LBL) patients who received dose-adjusted Berlin–Frankfurt–Münster (BFM)-90 regimen as first-line therapy in our center. A total of 145 T-LBL patients who underwent first-line dose-adjusted BFM-90 was retrospectively reviewed. Conditional survival analysis was used to evaluate the long-term prognosis of patients. Receiver operating characteristic (ROC) curve was applied to determine the optimal cut-off value for neutrophil-to-lymphocyte ratio (NLR). Estimated 3-year overall survival (OS) and progression-free survival (PFS) rates for overall were 66.8% and 58.4%, respectively. Conditional survival analysis showed that for patients having survived 3 and 5 years or more after the completion of the treatment, the estimated subsequent 3-year OS thereafter increased to 85.7% and 94.3, respectively. Patients receiving consolidation APBSCT (Autologous peripheral blood stem cell transplantation) after BFM-90 regimen had superior 3-year OS than those with non-APBSCT (79.1% *vs*. 33.4%, *p*<0.001). We also discovered that baseline NLR ≥4.95 was negatively associated with OS (HR=2.75, 95% CI 1.55-4.89, *p*=0.015) and PFS (HR=2.07, 95% CI 1.25-4.96, *p*=0.021) via multivariable analysis. Conclusions: The survival probability of T-LBL patients treated with first-line dose-adjusted BFM-90 has improved significantly as patients have survived for every additional year. The addition of consolidation APBSCT following dose-adjusted BFM-90 therapy bring further survival benefits for those patients. Baseline NLR ≥4.95 was an independent risk factor for T-LBL patients in our study.

## INTRODUCTION

T-cell lymphoblastic lymphoma (T-LBL) is a heterogeneous malignancy derived from T-cell lineage which is more common in teenagers and males. It accounts for 3.4% of all cases of Non-Hodgkin lymphoma (NHL) in China [1]. T-LBL patients usually exhibit signs of bone marrow (BM) invasion. Central nervous system (CNS) involvement is a frequent site of relapse in the absence of CNS prophylaxis [2]. T-LBL and T cell acute lymphoblastic leukemia (T-ALL) have important cytological and histological features in common [3]. The distinction between these two entities is determined by the percentage of BM involvement: T-LBL patients have less than 25 % lymphoblast in bone marrow, whereas patients with more than 25 % bone marrow replacement are classified as having T-ALL. The prognosis of T-LBL has improved considerably with the application of ALL-type therapy, such as Berlin–Frankfurt–Münster (BFM) regimens [4–8]. To further reduce adverse effects associated with BFM regimen, dose-adjusted approaches have been attempted recently, including BFM-90 [7–9].

Apart from first-line chemotherapy, APBSCT or maintenance therapy with 6-mercaptopurine and methotrexate following chemotherapy has been suggested for the treatment of hematological malignancies to further improve the prognosis of patients [10–12]. Patients with different types of lymphoma have been shown to benefit from APBSCT [13, 14]. In our previous study of 57 T-LBL patients receiving BFM-90 regimen, no significant differences in OS and PFS were observed between patients following APBSCT or not [8]. However, in that study, the follow-up period was comparably short with a median follow-up of 24 months, which might negatively influence the survival benefit of additional APBSCT. Besides, maintenance treatment in T-LBL patients has not been analyzed in that study. Conclusively, it is unclear whether APBSCT and maintenance following dose-adjusted BFM-90 could benefit T-LBL patients in large cohort with long-term follow-up.

Time-dependent analysis could reflect real-time changes in survival or risk at a given time point. Conditional survival (CS) as a statistical method was used widely to evaluate the survival of patients having survived beyond certain time. CS can produce more meaningful prognosis information for cancer patients than OS estimating from the time of treatment, especially for cancer types that can be cured [15, 16]. From CS analysis, clinicians could predict the long-term survival probability of patients. At present, data showing CS of T-LBL after treatment are lacking. Considering the promising outcomes of T-LBL patients treated with dose-adjusted BFM-90 followed by APBSCT or maintenance treatment, the analysis of long-term survival probability using CS for these patients could be critical.

Therefore, in the present study, we aimed to use conditional survival analysis to evaluate the long-term treatment outcomes of dose-adjusted BFM-90 therapy followed by APBSCT and or maintenance treatment. T-LBL patients have diverse prognoses, even with the same first-line regimen, thus we intend to explore whether there are some baseline biological characteristics that could predict the response and survival of dose-adjusted BFM-90 of T-LBL patients.

## RESULTS

### Patient characteristics

Eventually, 145 patients with newly diagnosed T-LBL who were treated with a dose-adjusted BFM-90 regimen in Beijing Cancer Hospital between 2004 and 2019 were included into our study. Patient baseline characteristics were listed in Table 1. The median age was 26 (range, 8–69) years, and there were 22.1% pediatric patients included in our study (age≤18, 32/145). Staging was carried out according to the Ann Arbor system for NHL in our current study. Most (70.3%) patients were males and presented with stage-III or stage-IV disease (122/145, 84.1%). CNS invasion was detected in 10 (6.9%) patients, and BM infiltration was found in 70 patients (48.3%) with 29 cases having >25% lymphoblast in the BM. Of all 145 T-LBL patients, 102 (70.3%) had mediastinal invasion at the initial diagnosis and, among these patients, 42 (41.2%) cases presented with a mediastinal mass of diameter ≥7.5 cm. Fifty-two (35.9%) cases and 93 (64.1%) patients were in International Prognostic Index (IPI) group 0–1 and IPI group 2–4, respectively.

**Table 1:** Patients’ characteristics and prognostic factors for OS and PFS.

**Variable**	**Number (%)**	**3-year OS (%)**	**p value**	**3-year PFS (%)**	**p value**
**Gender**					
Male	102 (70.3)	67.10	0.863	60.80	0.749
Female	43 (29.7)	62.00		58.10	
Age					
≤18	32 (22.1)	64.80	0.500	52.30	0.222
≥18	113 (29.7)	69.30		59.90	
**Stage**					
I+II	23 (15.9)	94.40	0.011	85.60	0.028
III+IV	122 (84.1)	60.60		53.70	
**Bulky mass(>7.5cm)**					
Yes	49 (33.8)	60.90	0.715	63.00	0.458
No	96 (66.2)	57.70		69.50	
**Ki67**					
≥75%	80 (55.2)	61.20	0.089	60.40	0.571
<75%	53 (36.5)	73.10		55.90	
**LDH**					
normal	87 (60.0)	71.80	0.104	63.00	0.360
abnormal	58 (40.0)	57.00		52.20	
**CNS involvement**					
Yes	10 (6.9)	22.00	0.002	9.40	<0.001
No	132 (93.1)	69.30		62.50	
**BM involvement**					
Yes	70 (45.5)	57.40	0.003	48.70	0.012
No	69 (49.0)	78.70		71.10	
**Mediastinal invasion**					
Yes	102 (70.3)	66.30	0.925	52.00	0.088
No	43 (29.7)	67.50		60.90	
**Response**					
CR	85 (58.6)	80.00	<0.001	74.10	<0.001
PR	39 (26.9)	61.30		56.40	
SD	15 (10.3)	58.20		18.20	
PD	6 (4.1)	0.00		0.00	
**B symptoms**					
Yes	45 (31.0)	58.60	0.106	54.10	0.317
No	100 (69.0)	69.40		61.10	
**ECOG**					
0	136 (93.8)	69.50	<0.001	61.60	<0.001
1	9 (6.2)	14.80		16.70	
**Extra nodal site**					
<2	66 (45.5)	77.90	0.017	67.90	0.017
≥2	79 (54.5)	55.90		50.80	
**IPI Score**					
0-1	52 (35.9)	84.10	0.001	71.70	0.005
2-4	93 (64.1)	56.10		49.20	
**Local Radiation**					
Yes	10 (6.9)	55.60	0.546	58.30	0.780
No	135 (93.1)	67.70		58.40	
**TBI**					
Yes	15 (27.3)	100.00	0.172	93.30	0.235
No	40 (72.7)	82.50		72.50	

LDH, lactate dehydrogenase; CNS involvement, Central nervous system; BM, bone marrow; CR, complete response; PR, partial response; SD, stable disease, PD, progressive disease; ECOG, Eastern Cooperative Oncology Group; IPI, International Prognostic Index; TBI, total body irradiation.

### Survival and conditional survival

**Figure 1:**
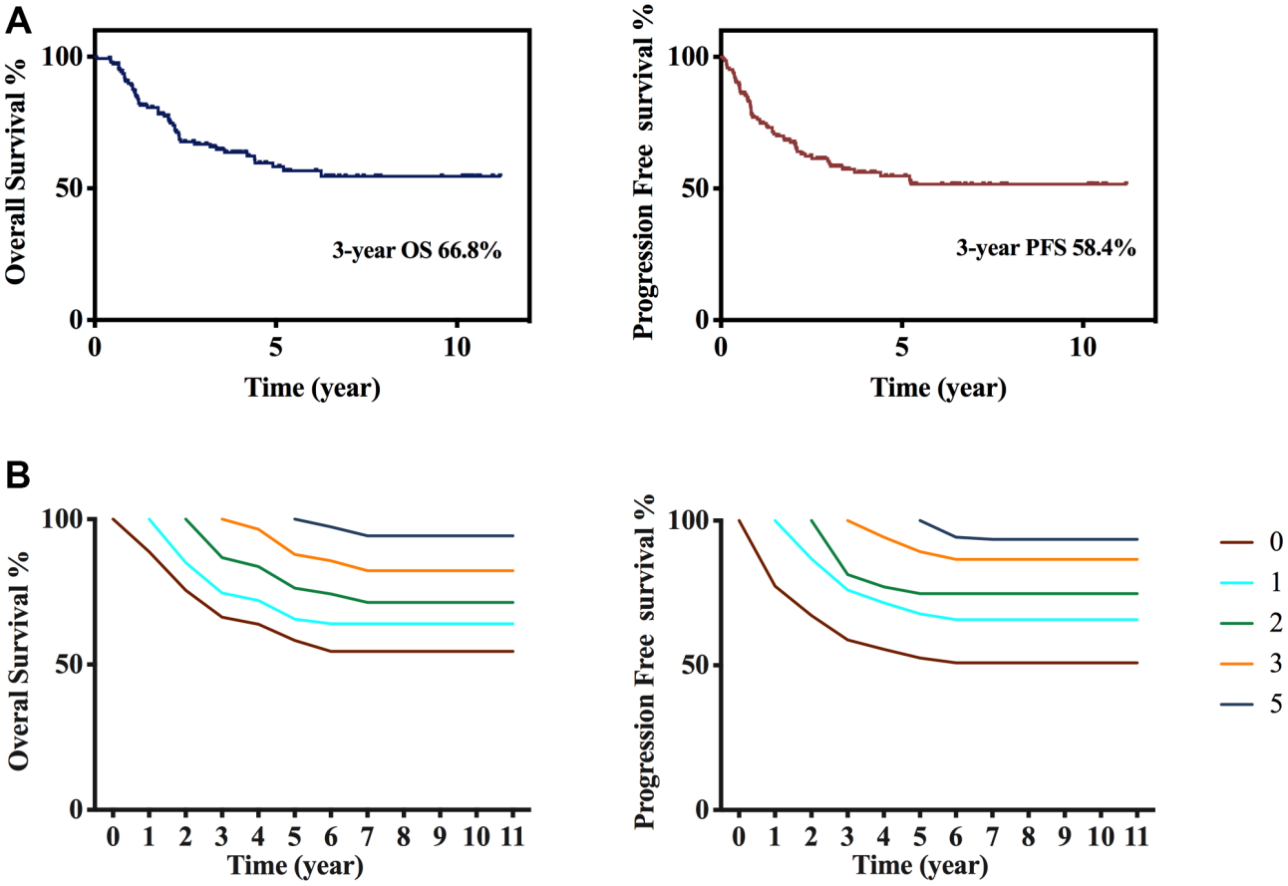
**Survival and conditional survival curves.** (**A**) Overall survival and progression free survival after diagnosis. (**B**) Overall survival and progression free survival conditional on having survived 1, 2, 3, and 5 years after treatment in total 145 patients.

The median duration of follow-up was 34.2 (range, 0.7–136.1) months. Till the end of last follow-up, 94 (64.8%) patients were alive. 3-year OS and PFS rates for the entire cohort were 66.8% and 58.4%, respectively (Figure 1A). Considering the long-time span of this study, we also compare the survival differences between different times. No significant changes in patients’ survival in different time zones were observed in our study cohort (Supplementary Figure 1). Conditional survival cures for T-LBL patients reveal a gradual increase in relative survival probabilities for every additional year. For patients who receiving dose-adjusted BFM-90 therapy, relative 3-year OS climbed up with each additional year: from 66.8% to 72% for 1-year survivorship, to 76.3% for 2-year survivorship, to 85.7% for 3-year survivorship and to 94.3% for 5-year survivorship. Similarly, 3-year PFS increased from 58.4% to 71.4%, 74.8%, 84.6% and 93.5%, respectively (Figure 1B).

### Value of APBSCT and maintenance treatment in T-LBL

**Figure 2:**
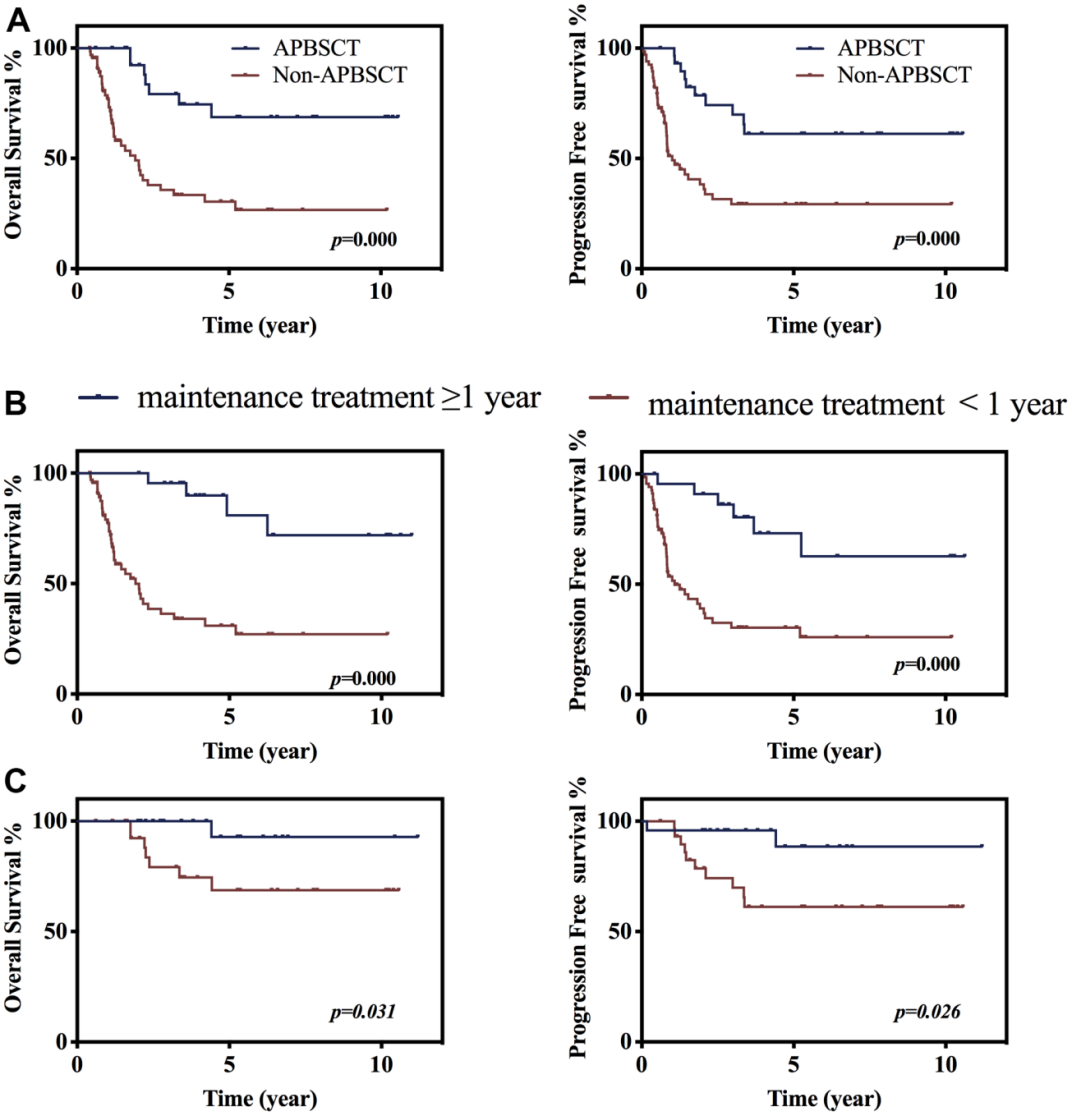
**The value of APBSCT and maintenance treatment following chemo-therapy on prognosis.** (**A**) Kaplan–Meier survival curve stratified by receiving APBSCT or not of patients without following maintenance treatment. P<0.001 (**B**) The impacts of maintenance treatment without APBSCT on overall survival and progression free survival. (**C**) The effects of maintenance treatment following APBSCT on overall survival and progression free survival.

APBSCT was indicated for patients who had a response to the dose-adjusted BFM-90 regimen. In this cohort, 54 patients had APBSCT after dose-adjusted BFM-90. Of these 54 patients, 24 patients also had following maintenance treatment ≥1 year, and among 91 patients without APBSCT treatment, 24 patients had maintenance treatment ≥1 year. To exclude the effects of maintenance treatment, we analyzed the remaining 30 with APBSCT and 67 patients without APBSCT. We discovered that APBSCT improved the prognosis considerably at 3 years compared with those who did not have APBSCT (OS, 79.1% *vs*. 33.4%; PFS, 69.9% *vs*. 29.2%, *p* < 0.001) (Figure 2A). TBI conditioning therapy before APBSCT was administered since the middle of 2016 in Beijing Cancer Hospital, and the median duration of follow-up after APBSCT was only 1.2 (range, 0.39–2.65) years. Fifteen cases of total 54 APBCST treated patients received TBI as conditioning therapy. Among these 15 patients, only one case had disease progression at 2.58 years after APBSCT, and no patient died during follow-up. However, we did not find significant differences between the TBI group and non-TBI group with respect to OS or PFS (Supplementary Figure 2A).

Maintenance treatment was suggested for patients, in our study, 48 patients had at least 1-year maintenance treatment. Maintenance treatment for ≥1 year for patients after dose-adjusted BFM-90 improved the prognosis significantly at 3 years compared with that for patients who did not have maintenance treatment for ≥1 year (OS, 80.9% *vs*. 36.3%; PFS, 79.8% *vs*. 30.3%, *p* < 0.001) (Figure 2B). Following ≥1-year maintenance treatment of patients who had APBSCT, produced better OS and PFS than patients without having maintenance treatment ≥1year after APBSCT therapy (OS, 92.9% *vs*. 79.1%; PFS, 88.5% *vs*. 69.9%, *p* < 0.05, Figure 2C). To clearly describe the value of APBSCT and maintenance treatment, we compare the OS and PFS of patients receiving either APBSCT or at least one-year maintenance treatment after BFM-90. While, no significant differences on survival were seen between patients having maintenance treatment ≥1 year and those having APBSCT after dose-adjusted BFM-90 treatment (Supplementary Figure 2B). Multiple-factor analysis also showed that APBSCT and maintenance treatment were independent protective factors for OS and PFS (Supplementary Table 1).

### Prognostic value of baseline factors

**Figure 3:**
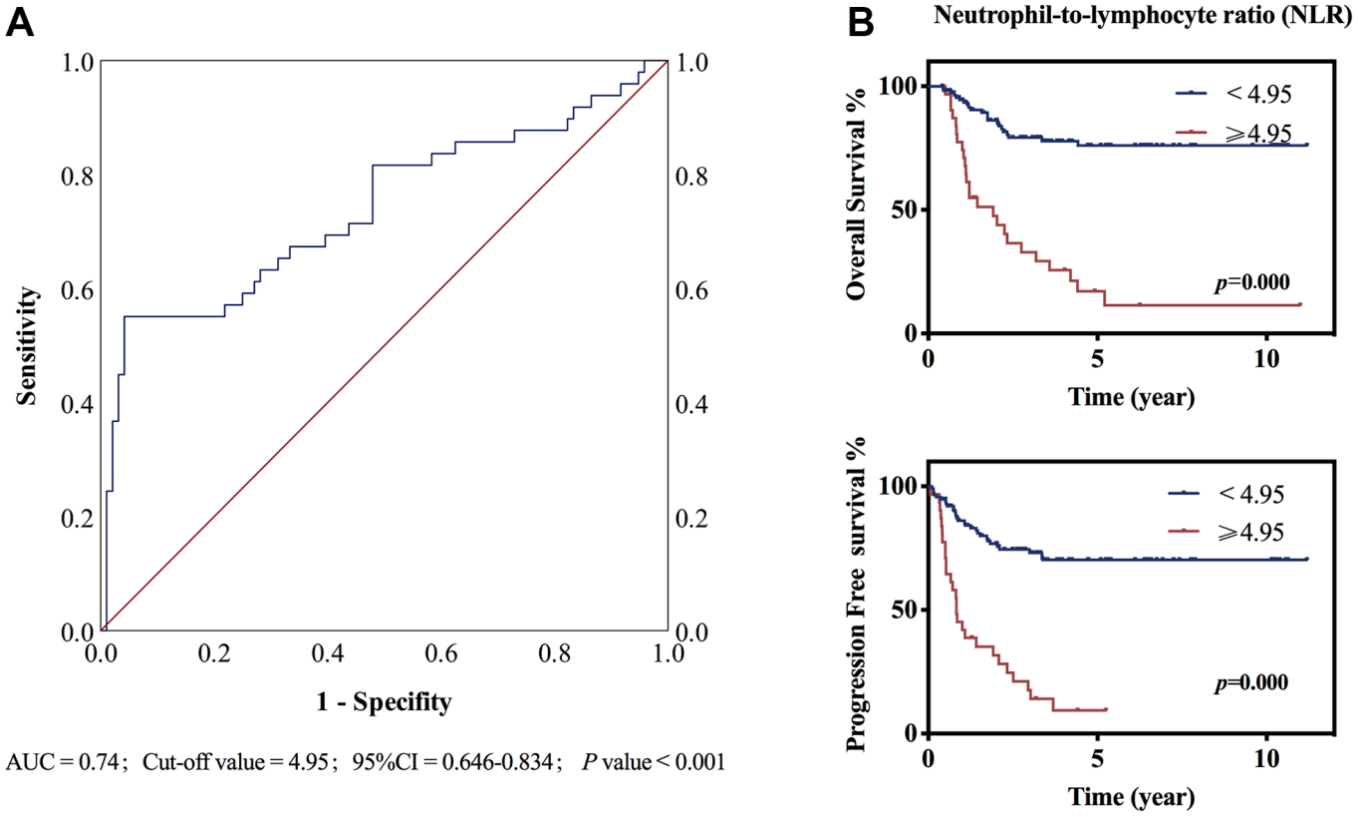
**ROC curve and Kaplan–Meier survival curve stratified by NLR.** (**A**) The ROC curve of NLR, the area under the ROC curve (AUC) was 0.74. p<0.001 (**B**) Neutrophil-to-lymphocyte ratio (NLR) ≥4.95 or <4.95 at diagnosis predicted overall survival and progression free survival. P<0.001.

Prognostic analysis of patients’ characteristics showed that IPI was a significant marker predicting prognosis of T-LBL patients receiving dose-adjusted BFM-90. Individuals with IPI of 0–1had better OS and PFS than those of 2–4 (3-year OS 84.1% *vs*. 56.1%; 3-year PFS 71.7% *vs*. 49.2 %, *p* < 0.01). CNS or BM involvement was adverse factors causing inferior OS and PFS (*p*<0.001). (Table 1) In addition to these traditional factors, immune cell parameters were reported with significant prediction function for survival [17]. However, there was no definition of the cut-off value for NLR, ranging from 2 to 7 in most studies [18–22]. In our study, we used OS as the endpoint of interest and ROC analysis was performed to calculate the optimal cut-off value for NLR. The area under the ROC curve (AUC) for NLR was 0.74, and the optimal cut-off values corresponding to the maximum joint sensitivity and specificity were 4.95 (p<0.001) (Figure 3A). Patients with NLR ≥4.95 had poor OS and PFS (p<0.001). (Figure 3B) Univariate and multivariate analysis also presented that NLR ≥4.95 was an independent risk factor for patients’ OS and PFS with hazard ratio at 2.39 (95% CI 1.55-4.89, *p*=0.015) and 2.07 (95% CI 1.25-4.96, *p*=0.021) respectively (Supplementary Table 1).

### Toxicity

Though adverse effects were common for most patients, there was no treatment-related death in patients treated with dose-adjusted BFM-90 in our cohort. Of 145 patients, 14 cases terminated treatment due to intolerable adverse events. The most common toxicity was myelosuppression, with 95 cases (65.5%) presenting with grade 3 or 4. Among these 95 patients, febrile neutropenia was seen in 24 patients. Also, 3.4% and 4.1% of cases presented with grade 3 liver injury and gastrointestinal-associated events, respectively. Four patients developed acute pancreatitis. Three patients suffered from acute renal failure due to administration of high-dose methotrexate.

## DISCUSSION

To our knowledge, this study was a comparably large-sample analysis of first-line dose-adjusted BFM-90 in treating T-LBL with a long-term follow-up period among Chinese population. Using conditional survival analysis, we further analyzed the treatment long-term outcomes of survivors in series. Moreover, we also demonstrated the encouraging survival benefit of adding APBSCT and maintenance treatment to first-line BFM-90 in T-LBL. In addition, our study identified NLR at the cut-off value of 4.95 as a new risk factor for evaluating survivals in T-LBL patients.

Our results showed that BFM-90 was one effective and tolerable treatment option with 3-year OS and PFS rates of 66.8% and 58.4%, respectively. Our data are comparable with results from other scholars [23]. Moreover, the dose-adjusted BFM-90 regimen was tolerable in our study: ~90% of patients completed treatment. Toxicity-related death was not seen in any patients. These data of this regimen safety are in accordance with results from other studies [3, 6, 24]. T-LBL is a curable disease, but the long-term survivals of patients suffering from T-LBL differs. CS has been reported for several hematological malignancies, and has provided information on the long-time prognosis [25–27]. CS analysis could provide information and evidence for clinicians to predict patients’ long-term prognosis. To analyze the long-term outcomes of T-LBL patients, we first described CS in patients who received dose-adjusted BFM-90 therapy in Beijing Cancer Center. We discovered that, after having survived for additional years, patients had a better prognosis and the 3-year OS increased steadily. Thirty-eight patients survived >5 years in this cohort, for these cases, at a median duration of follow-up of 78 (range, 54–136) months, only two cases died (one died due to cancer and one died of other reason). (Data not shown) Further analyses of the cause of death could provide long-time recommendations for the follow-up of patients.

In our study cohort, APBSCT was suggested for patients who responded to the dose-adjusted BFM-90 regimen, compared with patients not receiving APBSCT, patients having APBSCT treatment had better survival (p < 0.001), which was comparable with the previous studies [28, 29]. To further reduce the recurrence risk, maintenance therapies have also been proposed as a strategy against lymphomas [10–12]. It has been reported that maintenance therapy with 6-mercaptopurine and methotrexate after chemotherapy for ≥1 year could improve the prognosis of T-LBL patients, whereas benefits were not seen in children or adolescents with stage-III–IV T-LBL using maintenance chemotherapy regimens [30]. In our cohort, we discovered that maintenance treatment for ≥1 year after dose-adjusted BFM-90 therapy could significantly improve the 3-year OS and PFS compared with that not having maintenance treatment for ≥1 year (p < 0.05). Hence, we could recommend maintenance treatment for patients if permitted, and patients could benefit from maintenance treatment for ≥1 year irrespective of whether they underwent APBSCT or not.

Immune cell parameters were observed with significant prediction function for survivals in many cancer types. It has been reported recently the high NLR value probably was a biological marker for high-risk patients [18], and patients with high initial NLR had poor response to immune or chemo-therapy in solid cancers [19–22]. However, there was no definition of the cut-off value of NLR, ranging from 2 to 7 in most studies. In our study, we defined the optimal cut-off value of NLR at 4.95 using ROC curve analysis in T-LBL patients. Poorer OS and PFS were observed in patients with NLR ≥4.95 than those with NLR<4.94 (*p*<0.001), which was in accordance with the knowledge that high NLR was a risk factor for cancer patients [18]. Moreover, we for the first time reported the significance of NLR in predicting the prognosis of T-LBL patients receiving dose-adjusted BFM-90 therapy. But large-scale patients should be included to further verify whether NLR ≥4.95 was the optimal cut-off value for predicting patients’ survival.

Strengths of this study include dose-adjust regime and long duration of the study. We define the cut-off value of NLR at 4.95 in our center for predicting T-LBL patients’ survival for the first time. And, this is also the first study reporting on CS after first-line treatment for T-LBL. The data for CS can be utilized readily to provide information to T-LBL patients and physicians in clinical settings. From our current study, we discovered the significant value of APBSCT and maintenance treatment for T-LBL patients treated with dose-adjusted BFM-90 regimen. This information is important to both clinicians and patients, and it can help establish or strengthen treatment recommendations. Although T-LBL patients treated first with dose-adjusted BFM-90 over a 15-year period in Beijing Cancer Center were included, the sample size was relatively small. A further prospective study should be undertaken to provide more information on the dose-adjusted BFM-90 therapy employed in our study.

## CONCLUSIONS

T-LBL patients treated with dose-adjusted BFM-90 had promising outcomes with tolerable adverse effects. Conditional survival estimates illustrate improved survival probability for patients with initial survivorship. APBSCT and maintenance treatment could further prolong patients’ survival. Patients with a high initial neutrophil-to-lymphocyte ratio (NLR) ≥4.95 had poor outcomes.

## MATERIALS AND METHODS

### Study design and patient selection

The study cohort was derived from T-LBL patients receiving first-line BFM-90 therapy at Beijing Cancer Hospital between 2004 and 2020. Eligibility criteria were follows: (1) pathologically diagnosed with T-LBL according to World Health Organization classification of hematological malignancies; (2) patients receiving at least two cycles of first-line BFM-90 therapy; (3) at least one measure lesion. Exclusion criteria: (1) patients receiving prior systemic chemotherapy; (2) patients receiving other first-line regimens; (3) whose record was not intact or assessable. Declaration of Helsinki’s principles with approval by the Ethics Committee of the Beijing Cancer Hospital. (Beijing, China). Patients or their guardians provided written informed consent.

A total of 145 patients were included.

### Treatment and response assessment

All eligible patients received dose-adjusted BFM-90 regimen, which was derived from the BFM-90 regimen, but the specific frequency and dose of administration of cytarabine and methotrexate were reduced as we have previously reported [8]. In this study, for patients achieving partial remission (PR) or complete remission (CR) after administration of BFM-90 regimen, subsequent APBSCT was recommended if their physical status enabled them to. In addition to APBSCT, maintenance treatment with 6-mercaptopurine and methotrexate after chemotherapy or APBSCT was also suggested to consolidate the efficacy of patients who were response to BFM-90 regimen. Four patients (2.8%) with mediastinal mass also received radiotherapy in this study cohort.

Responses were assessed according to PET-CT or CT diagnosis. Recurrence of disease is determined by biopsy or imaging examination such as PET-CT. Death due to disease progression is called relapse-related death.

### Statistical analysis

OS was calculated from the date of the diagnosis to the date of death from any cause or the last follow-up. PFS was calculated from the date of diagnosis to the date of disease progression, death from any cause or the last follow-up. Survival curves were created using the Kaplan–Meier method and were compared using the log-rank test. Cox regression was applied to analyze the prognostic factors affecting OS and PFS.

Conditional survival estimates were obtained using the Kaplan-Meier method for the sub-cohort that had already survived a given length (x) of time after treatment. If S(x) is the unconditional (traditional) survival probability at time x, and the conditional survival probability S at time y>x is S(y|x) =S(x+y)/S(x) [15, 16]. Because conditional survival can vary by temporal changes in patient characteristics and supportive care, we calculated 3-year conditional survival rates of patients having survived for 1, 2, 3 and 5 years, respectively. And we evaluated the estimated 5-year survival probabilities for 3-year survivors.

We used OS as the endpoint of interest and receiver operating characteristic (ROC) analysis was performed to calculate the optimal cut-off value for NLR. The maximum joint of sensitivity and specificity was defined as the optimal cut-off value in our study.

Statistical analyses were undertaken using IBM Statistics SPSS Version 22.0 (Armonk, NY, USA). A two-side *p*<0.05 was considered significant.

### Ethics statement

The studies involving human participants were reviewed and approved by The Ethics Committee of the Beijing Cancer Hospital.

## Supplementary Material

Supplementary Figures

Supplementary Table 1
