# Supplementary material for: Survival and prognostic analysis of T-cell lymphoblastic lymphoma patients treated with dose-adjusted BFM-90 regimen
Source: Aging (Albany NY). 2022 Apr 10;14(7):3203–15. doi: 10.18632/aging.204008 (PMC9037275; doi:10.18632/aging.204008)
Supplement: Supplementary Figures [file aging-14-204008-s001.pdf]

SUPPLEMENTARY FIGURES

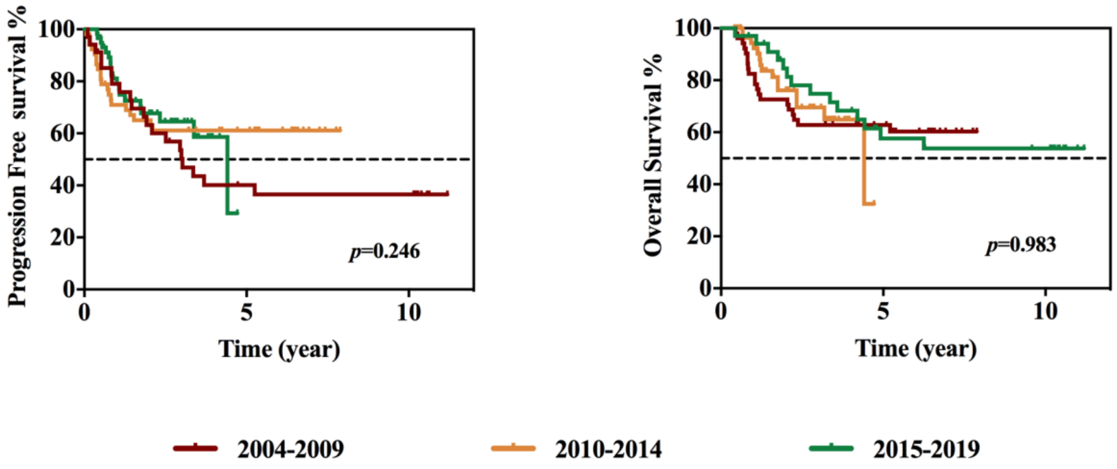

Supplementary Figure 1. Progression free survival and overall survival stratified by the year of diagnosis. Patients were divided into three groups (2004-2009, 2010-2014, 2015-2019) by the year of diagnosis.

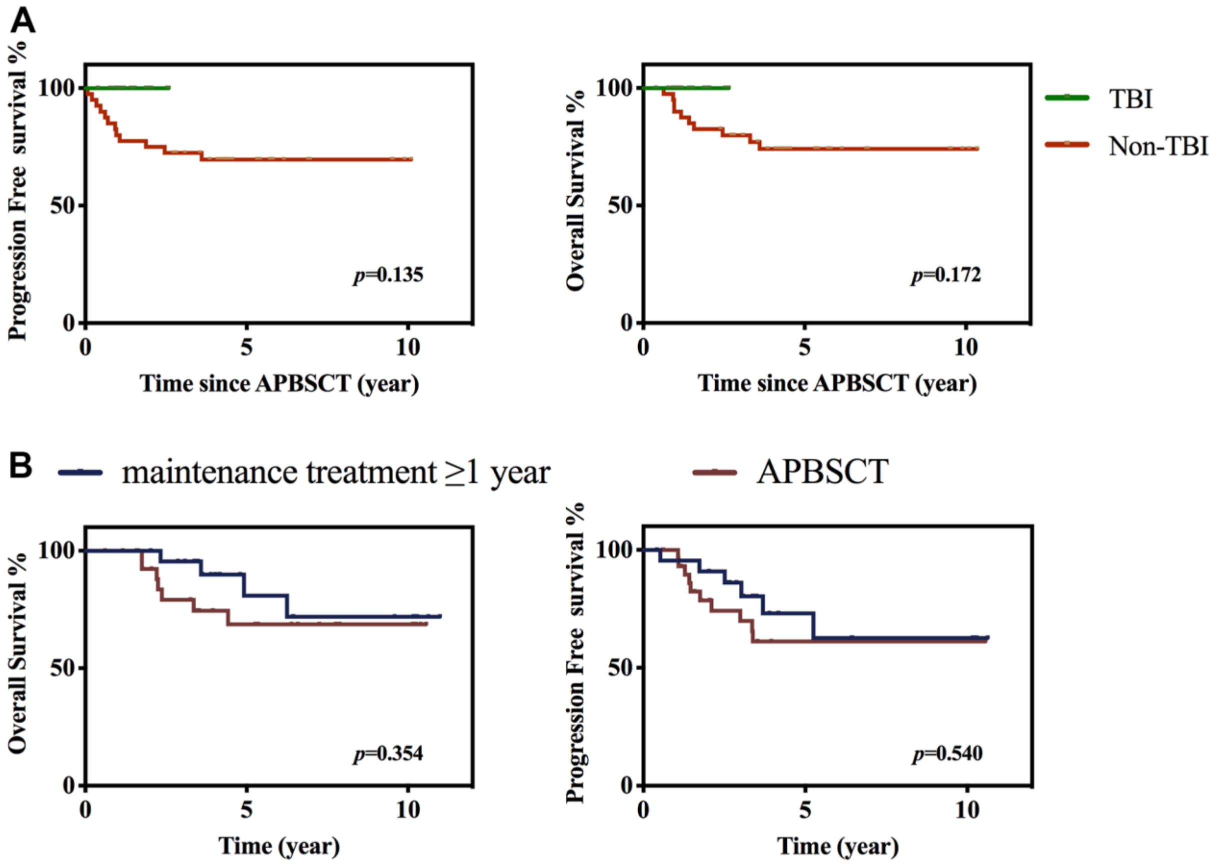

Supplementary Figure 2. (**A**) The impacts of conditioning regimen before APBSCT with TBI or not on patients' overall survival and progression free survival. (**B**) The comparison of subsequent APBSCT or maintenance treatment following dose-adjusted BFM-90 on patients' prognosis.
