# Supplementary material for: Survival and prognostic analysis of T-cell lymphoblastic lymphoma patients treated with dose-adjusted BFM-90 regimen
Source: Aging (Albany NY). 2022 Apr 10;14(7):3203–15. doi: 10.18632/aging.204008 (PMC9037275; doi:10.18632/aging.204008)
Supplement: Supplementary Table 1 [file aging-14-204008-s002.pdf]

SUPPLEMENTARY TABLE

Supplementary Table 1. Univariate and multivariate analysis of clinical parameters on OS and PFS.

	OS			PFS		
	**HR**	**95% CI**	**P-value**	**HR**	**95% CI**	**P-value**
**Univariate analysis**						
**The year of diagnosis**	1.06	0.53-2.09	0.878	0.62	0.33-1.16	0.133
	1.07	0.51-2.25	0.865	0.65	0.34-1.22	0.179
**Age (>18)**	1.24	0.66-2.35	0.508	1.43	0.80-2.54	0.238
**Gender**	0.96	0.51-1.82	0.910	1.07	0.61-1.88	0.818
**Stage (III/IV)**	3.30	1.03-10.63	0.040	3.10	1.12-8.55	0.029
**ECOG (>1)**	4.06	1.81-9.10	0.001	4.72	2.21-10.07	<0.001
**B symptom**	1.29	0.72-2.32	0.400	1.33	0.78-2.27	0.290
**BM involvement**	2.68	1.43-5.0	0.002	2.51	1.44-4.40	0.001
**Bulky mass (>7.5cm)**	1.41	0.80-2.49	0.250	0.95	0.55-1.64	0.857
**CNS involvement**	2.89	1.35-5.78	0.005	3.20	1.65-6.20	0.001
**Ki67 (≥75%)**	0.75	0.41-1.37	0.350	0.94	0.53-1.66	0.829
**Elevated LDH**	1.28	0.73-2.27	0.390	1.16	0.69-1.96	0.571
**Extra nodal site (>2)**	1.78	1.00-3.19	0.050	1.74	1.02-2.95	0.041
**IPI score (≥2)**	2.52	1.29-4.94	0.007	2.38	1.30-4.34	0.005
**Neutrophil-to-lymphocyte ratio (≥4.95)**	3.82	1.74-5.69	0.001	3.33	1.75-5.37	0.001
**APBSCT**	0.21	0.10-0.45	<0.001	0.27	0.14-0.50	<0.001
**Maintenance Treatment (≥1 year)**	0.13	0.05-0.33	<0.001	0.23	0.11-0.47	<0.001
**Multivariate analysis**						
**APBSCT**	0.18	0.08-0.40	<0.001	0.30	0.15-0.60	0.001
**BM involvement**	2.22	1.15-4.27	0.017	2.10	1.16-3.68	0.014
**CNS involvement**	2.97	1.37-6.43	0.006	3.50	1.70-7.21	0.001
**EC0G (>1)**	2.69	1.05-6.89	0.040	4.76	2.02-11.21	0.003
**Neutrophil-to-lymphocyte ratio (≥4.95)**	2.75	1.55-4.89	0.015	2.07	1.25-4.96	0.021
**Maintenance Treatment (≥1 year)**	0.16	0.06-0.40	<0.001	0.37	0.17-0.79	0.010

Factors with P<0.10 in the univariate analyses were subjected to multivariate analysis afterwards. Forward stepwise Cox proportional-hazard modeling was used in multivariate analysis of risk factors. LDH, lactate dehydrogenase; CNS, Central nervous system; BM, bone marrow; ECOG, Eastern Cooperative Oncology Group; IPI, International Prognostic Index; TBI, total body irradiation; APBSCT, autologous peripheral blood stem cell transplantation; The year of diagnosis was divided by every 5 years into three groups.
